# A Novel Fabrication Approach for Multifunctional Graphene-based Thin Film Nano-composite Membranes with Enhanced Desalination and Antibacterial Characteristics

**DOI:** 10.1038/s41598-017-07531-y

**Published:** 2017-08-08

**Authors:** Hanaa M. Hegab, Ahmed ElMekawy, Thomas G. Barclay, Andrew Michelmore, Linda Zou, Dusan Losic, Christopher P. Saint, Milena Ginic-Markovic

**Affiliations:** 1 Natural & Built Environments Research Centre, University of South Australia, Adelaide, SA 5095 Australia; 2 Institute of Advanced Technology and New Materials, City of Scientific Research and Technological Applications, Borg Elarab, Alexandria, Egypt; 3 Genetic Engineering and Biotechnology Research Institute, University of Sadat City (USC), Sadat City, Egypt; 4 School of Chemical Engineering, The University of Adelaide, Adelaide, SA 5005 Australia; 5 Centre for Pharmaceutical Innovation and Development, University of South Australia, Adelaide, SA Australia; 6 Future Industries Institute, University of South Australia, Adelaide, SA 5095 Australia; 7 Department of Chemical and Environmental Engineering, Masdar Institute of Science and Technology, Abu Dhabi, United Arab Emirates

## Abstract

A practical fabrication technique is presented to tackle the trade-off between the water flux and salt rejection of thin film composite (TFC) reverse osmosis (RO) membranes through controlled creation of a thinner active selective polyamide (PA) layer. The new thin film nano-composite (TFNC) RO membranes were synthesized with multifunctional poly tannic acid-functionalized graphene oxide nanosheets (pTA-f-GO) embedded in its PA thin active layer, which is produced through interfacial polymerization. The incorporation of pTA-f-GOL into the fabricated TFNC membranes resulted in a thinner PA layer with lower roughness and higher hydrophilicity compared to pristine membrane. These properties enhanced both the membrane water flux (improved by 40%) and salt rejection (increased by 8%) of the TFNC membrane. Furthermore, the incorporation of biocidal pTA-f-GO nanosheets into the PA active layer contributed to improving the antibacterial properties by 80%, compared to pristine membrane. The fabrication of the pTA-f-GO nanosheets embedded in the PA layer presented in this study is a very practical, scalable and generic process that can potentially be applied in different types of separation membranes resulting in less energy consumption, increased cost-efficiency and improved performance.

## Introduction

The use of reverse osmosis (RO) desalination technology is increasing yearly to produce the necessary freshwater resources for growing populations, with currently greater than 50% of desalination plants worldwide using RO technology^1–3^. Thin-film composite (TFC) membranes are the most prevalent membrane for RO and nanofiltration operations^4^, but suffer from trade-offs between both water flux and salt rejection as well as chlorine degradation and biofouling that leads to the loss of membrane flux and salt rejection performance^3, 5^. The increased requirements for membrane cleaning and replacement, as a result of biofouling leads to increases in RO operation costs. As such, biofouling of RO membranes remains one of the major challenges to be overcome to improve the efficiency of desalination^6, 7^. The biofouling is caused by microorganisms that adhere to the membrane surface under the hydrodynamic conditions of RO filtration^8^. This attachment is augmented by the non-specific physiochemical interactions among the bacterial cells and the membrane surface^9^, the occurrence of ligand/receptor interactions to membrane surface chemistry^10^ and the production of extracellular polymeric substances that intensify the adherence of bacterial cells^10, 11^. As such, RO membranes have been modified with biocidal agents, made with smoother surfaces to reduce the number of bacterial binding sites and produced with more hydrophilic chemistry to generate a hydrogen bonded hydration layer, helping to prevent bacterial attachment^12^.

As part of the efforts to address the challenges to RO technologies, a new type of thin film nanocomposite (TFNC) membrane was fabricated by incorporating zeolite nanoparticles within the polyamide (PA) active layer during the interfacial polymerization (IP) reaction^13, 14^. This resulted in the enhancement of the membrane properties in terms of the water flux and biocidal efficiency, without losing the desalination performance of the TFNC membrane. Even with this important development in TFNC membrane fabrication, challenges still remain. These include the poor dispersibility of such nanomaterials and consequently their aggregation within the PA active layer and the limitation of chemical interaction among nanomaterials and the PA active layer which is attributed to membrane defects. Therefore, more innovative research is still required to achieve TFNC membranes with high performance across all of the aspects of membrane filtration^15^.

Graphene nanosheets are another promising nanomaterial for RO TFNC membrane fabrication due to their smooth nature, superior biocidal activity and very large surface area^16, 17^, but their chemistry prevents dispersal in most solvents and interactions with other TFC components. Consequently, graphene nanosheets are oxidised to make graphene oxide (GO), covering the sheets with oxygenated functional groups, such as carboxyl, epoxide and hydroxyl groups^18^, that provide both convenient handles for chemical modification and better solvent dispersibility. Accordingly, GO holds promising potential to create a novel generation of GO-based TFNC membranes for water desalination application^17, 19^. Several researchers have investigated the improvement of antifouling and chlorine resistance performance of the TFC-RO membranes when GO/rGO was integrated within the PA thin active layer through the IP reaction^20–23^. However, these GO-based TFNC membranes generally suffered from a trade-off in which both water flux and salt rejection couldn’t be improved together. As one of the most important aims of membrane fabrication research has always been to tailor membranes that simultaneously improve flux and selectivity, more work is required to improve GO-based TFNC RO membranes.

To address the challenges of increasing both flux and selectivity, along with enhancing antibacterial characteristics of TFC membranes, a novel approach is presented in the current study, where TFNC RO membranes were fabricated with functionalized GO nanosheets embedded in the PA active layer. The GO nanosheets were first functionalized with tannic acid (TA) and polyethyleneimine (PEI), the TA tightly binding the GO and the PEI providing free amines available to be crosslinked both to the TA and into the active layer upon deposition of the PA. This crosslinking tightly integrates the GO into the TFNC, optimizing the robustness of the functional later. The fabricated membranes were subjected to a range of analyses to confirm their structure and chemical composition followed by evaluation of their water flux, selectivity, chlorine resistance and biocidal performance compared with pristine membranes enabling us to better understand the role of pTA-f-GO in improving TFC membrane performance.

## Results

### Surface characterization of the pristine and pTA-f-GO prepared membranes

#### TEM and AFM Analyses

**Figure 1:**
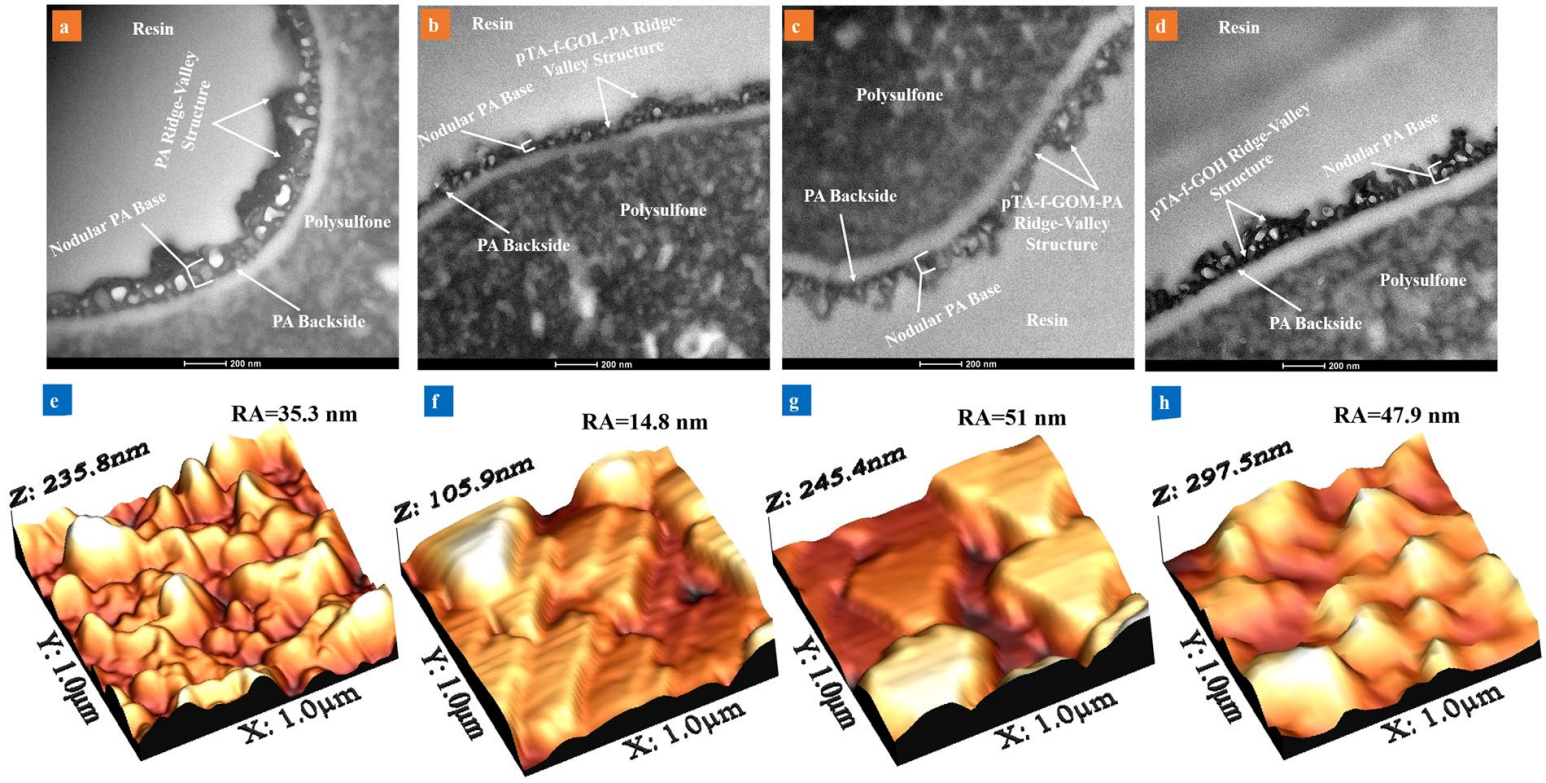
Cross-sectional TEM (scale bar of 200 nm) along with 3D AFM images of the prepared TFC membranes. The membranes were fabricated under the same conditions except the pTA-f-GO nanosheets content.

TEM and AFM analyses were performed to investigate the morphology and topography of the pristine and the pTA-f-GO embedded TFNC membranes (Fig. 1). The surface characteristics are important to filtration performance as the membrane surface roughness impacts the membrane antifouling properties^6, 7, 24^; increasing surface roughness both decreasing the efficiency of hydrodynamic cleaning processes^25^ and increasing in binding sites for bacterial cell attachment to the membrane surface^6, 25^. The TEM images illustrate changes in the cross-sections of membranes when the pTA-f GO nanosheets were embedded into the PA active layer. The thick and rough ridge and valley morphology of a typical polyamide coating was observed for the pristine membrane (Fig. 1a **)**. The incorporation of only 19 ppm of pTA-f-GOL nanosheets in the aqueous phase during the interfacial polymerisation generated a thinner, more compact layer, visually smoothing the PA structure (Fig. 1b). In contrast, integration of higher concentrations of pTA-f-GO resulted in the creation of thicker and rougher pTA-f-GO-PA active layers compared to pTA-f-GOL (Fig. 1c and d).

The membrane roughness average measured by AFM was 35.3 ± 3.8 nm for the pristine sample (Fig. 1e) and the GO-f-pTA-TFNC with low, medium and high contents had roughness average values of 14.8 ± 1.6, 51 ± 2.4 and 47.9 ± 3.2 nm, respectively (Fig. 1f–h). The results revealed that high concentrations of GO-f-pTA nanosheets in the TFNC increased the roughness of the membranes compared to pristine membrane, but that utilising low concentration of f-GO nanosheets significantly reduced roughness. This may be attributed to a highly dispersed solution of GO-f-pTA nanosheets in the aqueous phase^26^, allowing them to lie parallel to the membrane surface (Fig. 1f **)**.

#### ATR-FTIR analysis

**Figure 2:**
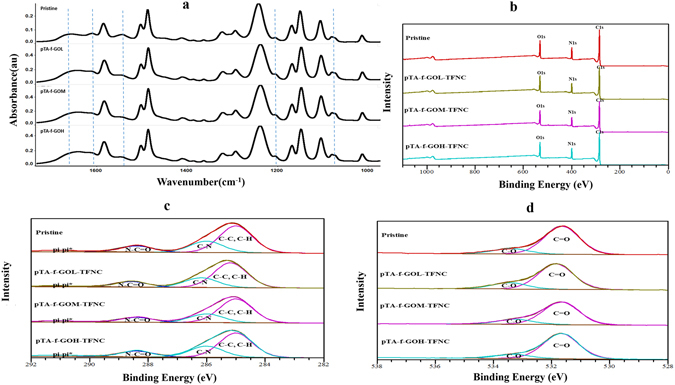
The surface chemistry profile including: FTIR spectra of pristine and pTA-f-GO based membranes (**a**), XPS survey scan profiles of pristine, pTA-f-GO based membranes (**b**) and C1s and O1s high resolution curves (**c** and **d**).

The ATR-FTIR spectra of the pristine and pTA-f-GO fabricated membranes are presented in Fig. 2a. The characteristic peaks of the thin PA active layer of the pristine membrane are at 1663, 1609, 1570, 1205 and 1090 cm^−1^ representing the amide I (C=O stretch), C=C stretching, amide II (N−H bending), C-O and C-C bending and stretching respectively, in addition to O–H bond stretching between 3000 and 3500 cm^−1^ (not shown)^7, 19^. In comparison, for the fabricated TFNC, the amide I (C=O stretch) peak was shifted to higher intensity and the other peaks changed in character, confirming the presence of pTA-f-GO nanosheets in the PA thin active layer.

#### XPS analysis

XPS analyses were performed to assess the surface chemical structure of the membranes including atomic composition, functional group content and degree of cross-linking at the surface of the PA active layer. Firstly, the sulphur peak of the Psf support layer disappeared from the XPS survey scan profiles of the fabricated membranes, confirming the successful formation of PA thin active layer on the top of Psf support layer for all membranes^27^. As shown in Fig. 2b, the XPS survey spectra of the pristine and pTA-f-GO fabricated membranes displayed the same elemental peaks, including C1s, N1s and O1s, at the membrane surface. Nevertheless, fractions of these elements (Table 1) are different due to the varying incorporation of pTA-f-GO nanosheets. For example, oxygen content is higher at the surface of the pTA-f-GOL.

**Table 1:** The elemental compositions, O/N ratio, chemical functional groups % and degree of cross-linking of the TFC, hence m + n = 1.

Sample	C (1s)	N (1s)	O (1s)	C-C, C-H	C-N	N-C=O	pi-pi*	O/N ratio	Cross-linking Density (%)
Pristine	75.7	11.5	12.8	56.4	23.9	13.3	6.5	1.11	84%
pTA-f-GOL-TFNC	75.4	11.3	13.3	58	23	13	5.6	1.17	76.5%
pTA-f-GOM-TFNC	76.3	10.9	12.7	59.5	21.8	12.5	5.3	1.16	78%
pTA-f-GOH-TFNC	75.8	12.1	12.1	55.6	25.3	13.5	5.6	1	100%

The C1s high resolution spectra **(**Fig. 2c **)** illustrated three peaks at binding energies of 285, 286 and 288.5 eV. These are assigned to a main peak of aliphatic and/or aromatic carbons (C-C and C-H), a peak that is attributed to carbon atoms attached to weakly electron withdrawing (C− N) and a minor peak that is associated with carbons attached to strongly electron withdrawing atoms including carbons in carbonyl groups (O=C−O and O=C−N)^28^. The O1s high resolution spectra also confirmed the presence of carbonyl groups (C=O) at the top of PA active layer with a peak at 531.6 eV (Fig. 2d), as well as a second oxygen type (C-O) at 533.2 eV.

**Figure 3:**
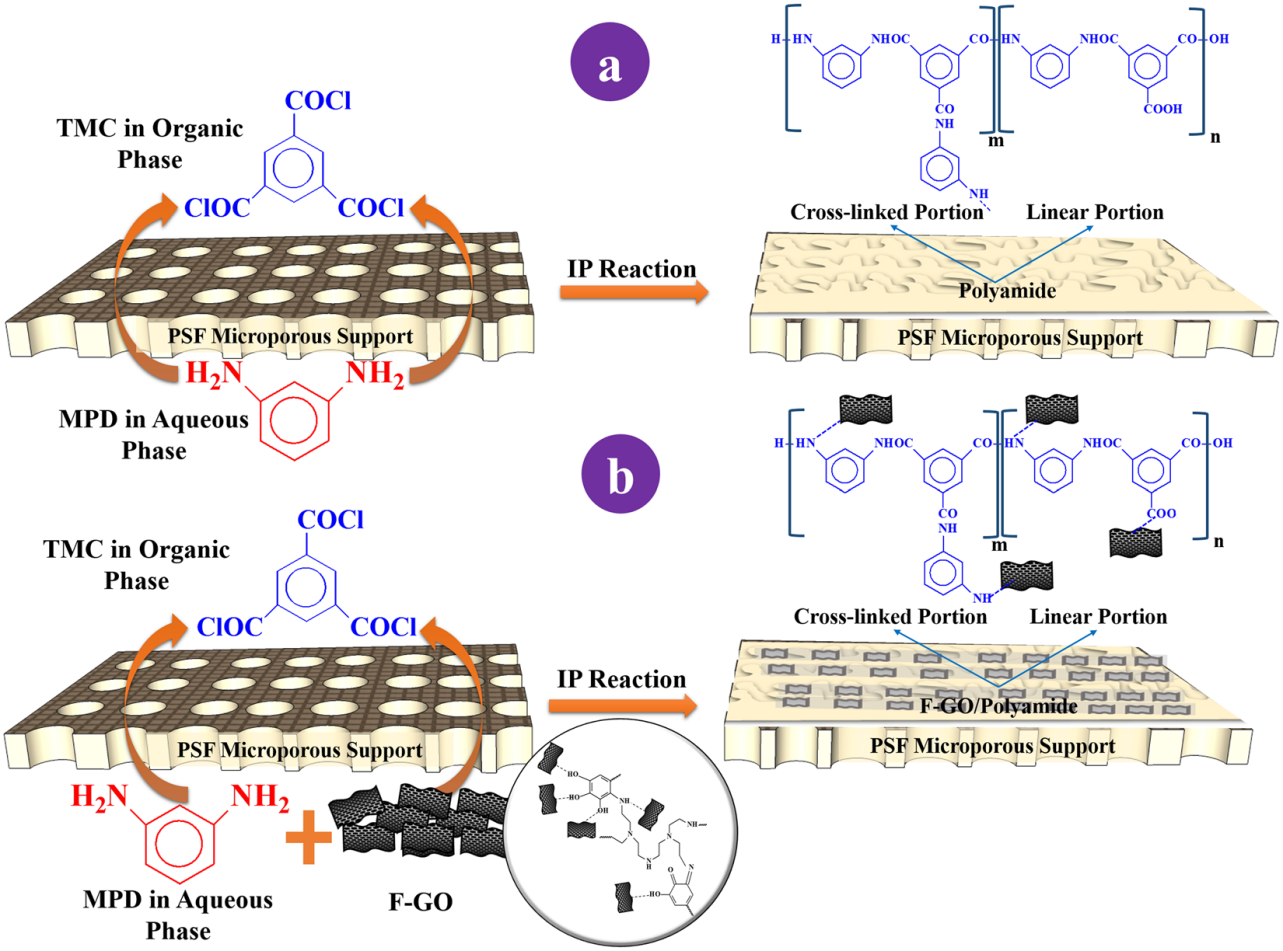
(**a**) Schematic depiction of IP reaction among MPD aqueous phase and TMC organic phase on the top of PSF microporous support and the chemical formulation of pristine PA layer, where m and n denotes the crosslinked and the linear portions of PA layer. (**b**) The chemical reaction and formulation of pTA-f-GO nanosheets embedded into the PA layer within MPD aqueous phase.

The elemental composition percentages on the membrane surface were evaluated by XPS analysis and used to calculate the cross-linking degree of the PA thin active layer through equations (1 and 2)^29, 30^, where *m* is the cross-linked portion and *n* is the linear part of the PA thin active layer as presented in Fig. 3.

1 $${\rm{Degree}}\,{\rm{of}}\,{\rm{crosslinking}}\,( \% )=\frac{{\rm{m}}}{{\rm{m}}+{\rm{n}}}\times {\rm{100}}$$

2 $$\frac{{\rm{O}}}{{\rm{N}}}=\frac{{\rm{3m}}\,+\,{\rm{4n}}}{{\rm{3m}}+{\rm{2n}}}$$

Hypothetically, O/N ratio fluctuates between 1 and 2, with 1 representing a fully cross-linked structure with a chemical formula of C_18_H_12_N_3_O_3_ (*n* = 1 and *m* = 0) and 2 representing a fully linear structure with a chemical formula C_15_H_10_O_4_N_2_ (*n* = 0 and *m* = 1). Table 1 displays the XPS analysis of the fabricated membranes. The results showed that increasing the content of pTA-f-GO nanosheets increased the cross-linking density of the fabricated TFC membranes from 76.5% to 100%.

#### Surface charge analysis

**Figure 4:**
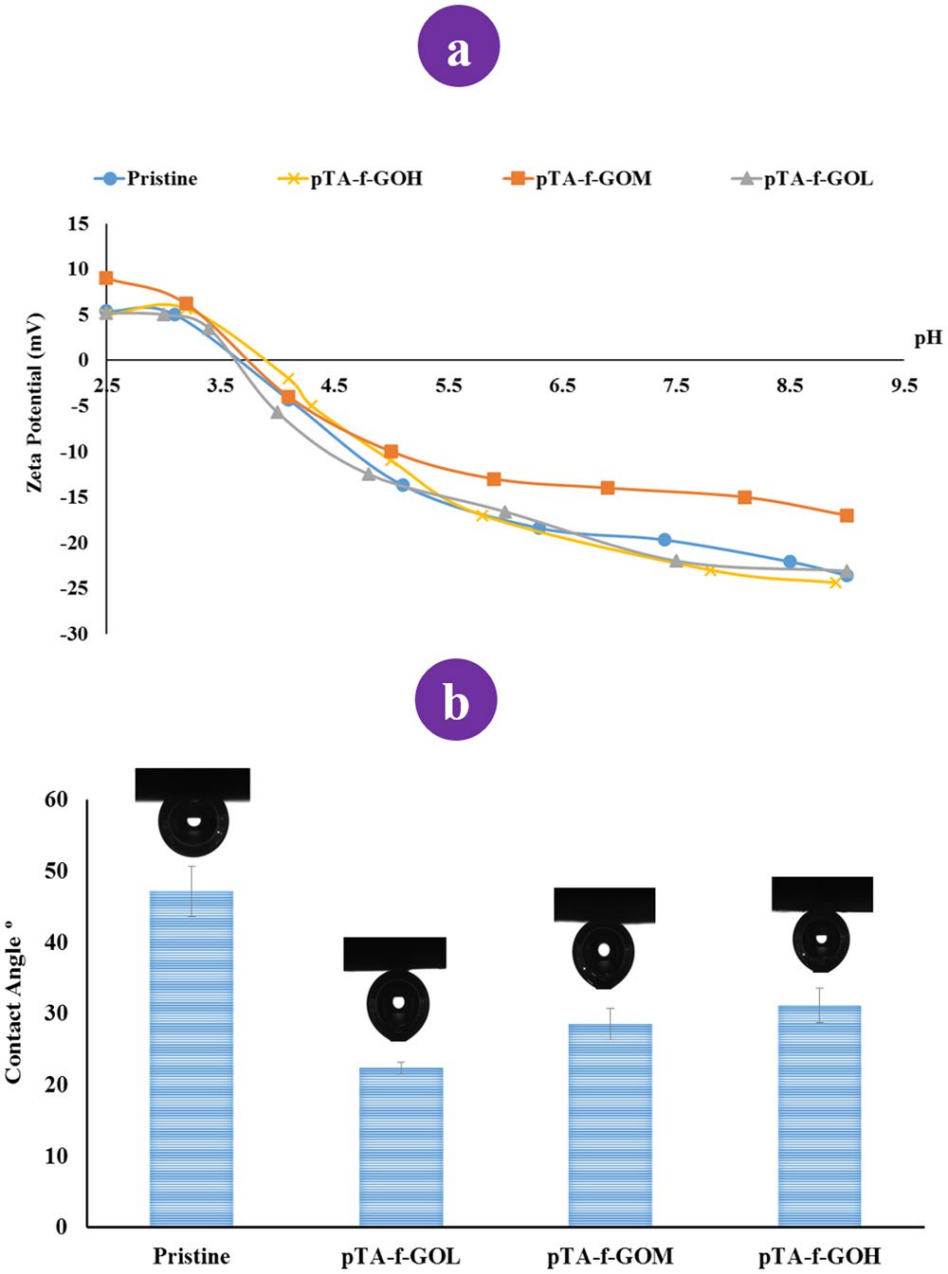
(**a**) Surface charge and (**b**) wettability measurements of pristine and pTA-f-GO RO TFNC fabricated membranes.

The zetapotential measurements of the prepared membranes were plotted against pH as shown in Fig. 4a. In general, all the membranes started with positive charge when exposed to acidic solutions and alter to negative charge with increasing pH, with both pTA-f-GOL and pTA-f-GOH membranes having very similar charge behaviour to the pristine membrane. In contrast, above pH 5, the pTA-f-GOM membrane has clearly less negative Zeta potential than the other membranes. This is attributed to the pTA-f-GOM membrane having less free acid groups^31^ and more phenolic and amine groups on its surface^32^.

#### Wettability analysis

There is a positive correlation between the membrane surface hydrophilicity and the enhancement of the antifouling properties of membranes^19, 24, 33^. The increase in membrane hydrophilicity results in reduced interfacial energy between the membrane surface and water molecules, leading to high water flux and reduced membrane fouling^34, 35^. Figure 4b illustrates the wettability profiles of pristine and pTA-f-GO membranes. The pristine TFC membrane has a PA active layer with a contact angle of 47° ± 3.53, which is significantly shifted to 22° ± 0.82 by the incorporation of low amounts of pTA-f-GO nanosheets in the PA layer **(**Table S3 **)**. The contact angle significantly increased to 28.5° ± 2.15 and 31° ± 2.45 as the pTA-f-GO nanosheets content increased in the PA layer.

### Assessment of the desalination performance of the membranes

**Figure 5:**
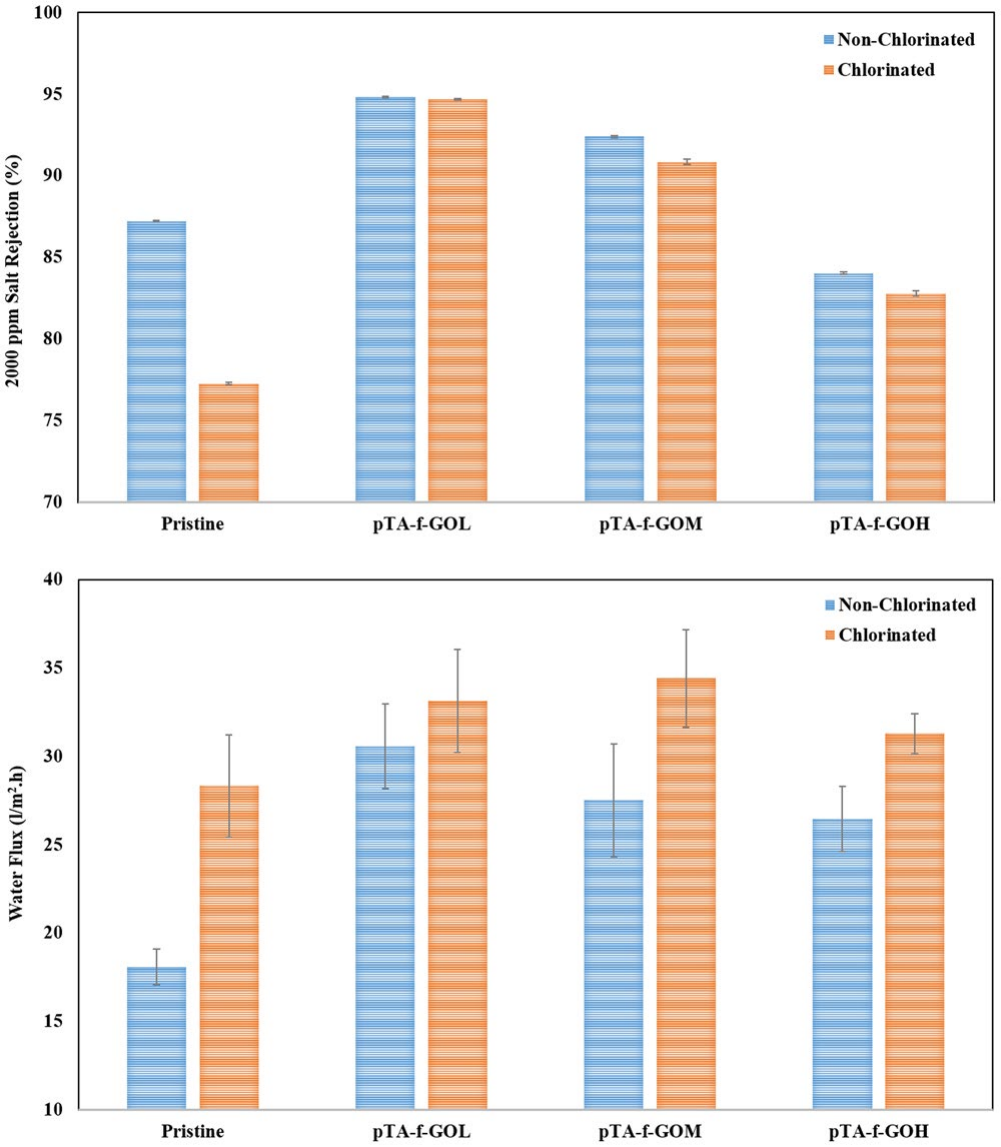
Water flux, salt rejection (%) and chlorine resistance performance of the pristine and fabricated membranes with various pTA-f-GO nanosheet loadings.

The desalination performance of the membranes in terms of water flux and salt separation was measured by filtering a NaCl solution (2000 ppm) at 250 psi using a high pressure stirred cell. The results indicate that the water flux of the pristine membrane was significantly (p < 0.05) lower than other membranes fabricated with pTA-f-GO nanosheets **(**Fig. 5, Tables S1 and S2 **)** with improvements in permeation compared to the pristine of 41%, 34% and 32% for the pTA-f-GOL, pTA-f-GOM and pTA-f-GOH fabricated membranes respectively. Salt rejection was also influenced by the pTA-f-GO membrane incorporation and followed a similar trend to the water flux results, with lower concentrations of pTA-f-GO significantly improving the selectivity compared to the pristine membrane **(**Fig. 5, Table S2 **)**. The improvement in the salt separation performance of pTA-f-GO membrane can be attributed to the enhancement of the physicochemical properties of the dense thin layer, providing a thinner and smother PA layer. This allows the fabricated pTA-f-GOL TFNC and pTA-f-GOM TFNC membranes to overcome the trade-off between the membrane water permeation and salt rejection.

After the chlorination, the variation in the salt rejection of the pTA-f-GO-TFNC membranes compared to the equivalent non-chlorinated membranes was minor. In contrast, the pristine membrane salt rejection decreased from 87% to 77% and water flux increased from 18 l/m^2^.h to 28 l/m^2^.h **(**Fig. 5 **)**, attributable to the degradation of the PA layer triggered by chlorine substitution^36^. Generally, the water flux and salt rejection of the pTA-f-GO fabricated membranes were significantly higher than those of the pristine and the performance of the pTA-f-GOL were not degraded by chlorination. The improved chlorine resistance of the pTA-f-GOL membrane could be related to the creation of intensive hydrogen bonding network among pTA-f-GO and PA without aggregation^37^. Moreover, the pTA-f-GO nanosheets can shield the underlying PA layer from chlorine degradation^38^.

### The effect of pTA-f-GO incorporation on the biocidal properties of membranes

**Figure 6:**
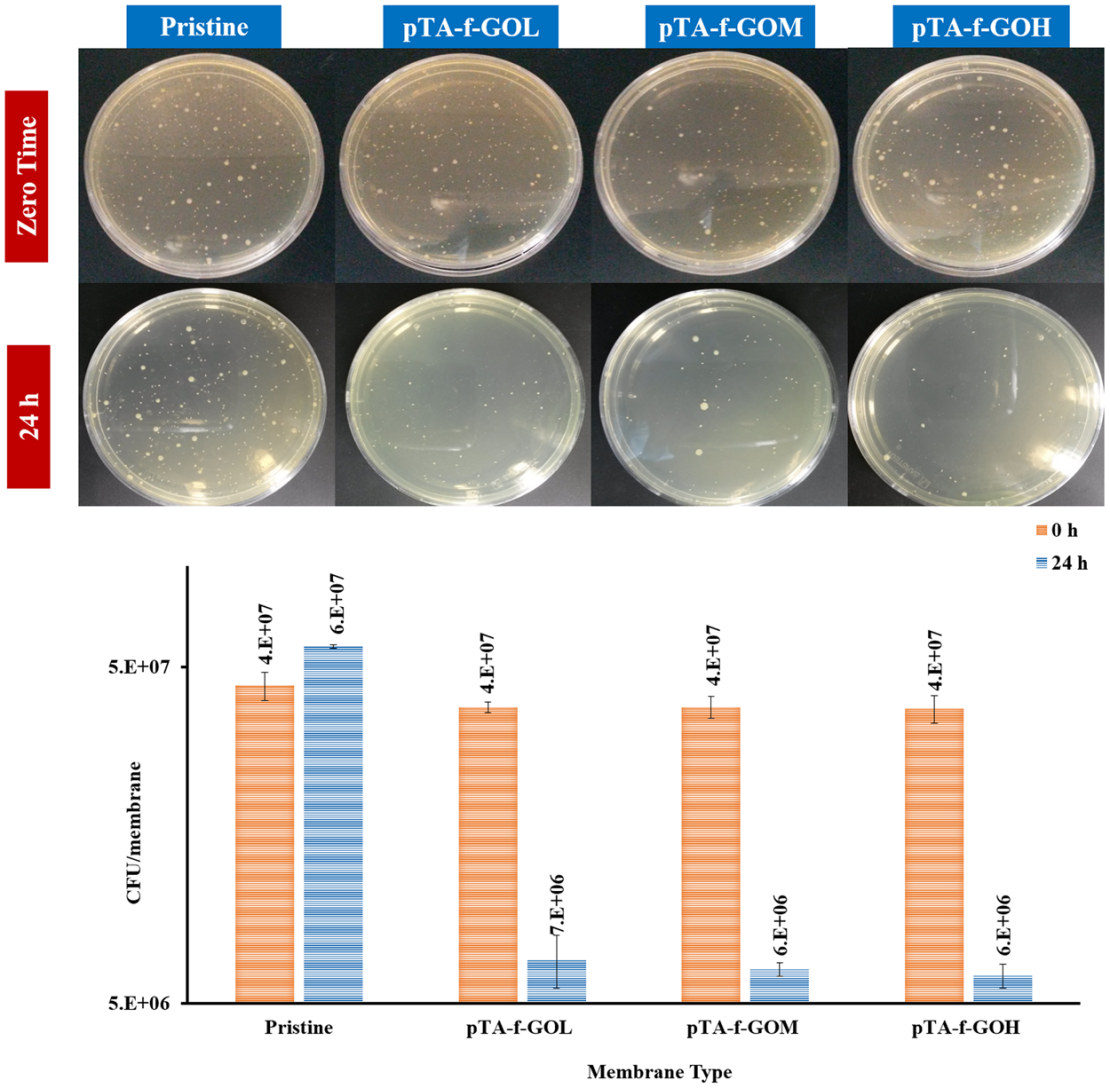
The biocidal activity of the pristine and pTA-f-GO TFNC fabricated membranes as a function of GO concentration.

The biocidal properties of pristine, pTA-f-GOL, pTA-f-GOM and pTA-f-GOH fabricated membranes were evaluated using the American Association of Textile Chemists and Colorists (AATCC) test method 100–2004 as presented in Fig. 6. The bacterial strain grew well on the pristine membrane at zero time and its growth was further enhanced after 24 h. Generally, all the pTA-f-GO membrane samples displayed a high bactericidal activity of at least 82% reduction in the bacterial colonies of *E. coli* compared to the pristine membrane, with the pTA-f-GOH membrane showing the highest antibacterial ability (83.8%). This can be attributed to the presence of f-GO that inhibits bacterial growth due to the presence of reactive oxygen species (ROS) along with its sharp edges^24, 39^. In addition, previously there has been shown to be a synergetic effect when combining pTA-pEI and GO, providing the fabricated pTA-f-GO membranes with their high biocidal effect following the capturing and killing mechanism^18^.

## Discussion

The surface nature of the PA thin active layer was crucially changed by the incorporation of pTA-f-GO, which is evident from contact angle, TEM and AFM results. This is attributed to the pTA-f-GO incorporation in the m-phenylenediamine (MPD) solution, leading to a variation in the MPD solubility in the interfacial reaction. In the case of the pristine membrane, the MPD molecules in the aqueous phase have a greater tendency to diffuse with faster migration rates into the organic solution containing trimesoyl chloride (TMC), which allows a high polymerization rate and the formation of a thicker PA active layer. In contrast, when low concentrations of pTA-f-GO were incorporated in the aqueous phase containing MPD, non-covalent interactions between the pTA-f-GO and MPD could slow the transport of MPD into the organic phase. This would slow the polymerization rate and better control the polymerization reaction compared to the pristine membrane, resulting in a thinner, smoother PA active layer that enhances water flux. In this case, the diffusion of water from the aqueous phase to the organic phase may become more significant as a competitive reaction compared to the pristine case, consuming more acid chloride groups of TMC and preventing as much crosslinking^40^. This is reflected in the lower crosslinking of pTA-f-GOL measured by XPS and the thinner PA active layer. Despite this, increasing the concentration of pTA-f-GO nanosheets for pTA-f-GOM and pTA-f-GOH in the aqueous phase with the MPD did not increase this effect. This could be attributed to the reactive groups on the pTA-f-GO becoming a more significant component of the reaction with increasing pTA-f-GO; TA able to react with both MPD and TMC and PEI able to react with TMS. As such, increasing pTA-f-GO beyond 19 ppm increased crosslinking, active layer thickness and roughness and reduced hydrophilicity. This ultimately resulted in lower salt rejection and water flux compared to the pTA-f-GOL membranes^41^. Combining the strong antibacterial results with the excellent filtration performance of the pTA-f-GOL shows that it can be practically utilized to fabricate robust TFNC membranes with high performance that mitigate against membrane biofouling and reduce total operational costs.

## Materials and Methods

Graphite powder SP-1 < 20 micron was purchased from Bay Carbon™ (USA). Nitric acid (70%), sulphuric acid (98%), hydrochloric acid (30%), acetic acid (99.7%), KMnO_4_ powder, sodium hypochlorite solution, NaCl, H_2_O_2_ (30%), tannic acid (TA), polyethyleneimine (PEI), bicine, potassium chloride, m-phenylenediamine (MPD), trimesoyl chloride (TMC), sodium dodecyl sulfate (SDS), triethylamine (TEA) and camphor sulfonic acid (CSA) were purchased from Sigma-Aldrich™. Polysulfone (Psf) ultrafiltration membranes from GE were used as substrate in this study.

### Preparation of pTA-f-GO TFNC membrane

GO nanosheets were synthesized by modified Hummers technique as in our previous report^7, 42^. The GO nanosheets were dispersed in water forming a suspension (2 g/l) and then coated with TA cross-linked with PEI^26^, described as pTA-f-GO. The Psf membrane was positioned on a polymethylmethacrylate plate with the active side facing up and sealed with a neoprene rubber gasket and a polymethylmethacrylate frame, the set-up held together using binder clips. The TFNC membrane was fabricated by exposing the active side of Psf support layer for 5 min to an aqueous solution of MPD (2 wt%), TEA (2 wt%) and CSA (2 wt%) containing a series of different concentrations of pTA-f-GO nanosheets (0 to 76 ppm; dispersed with ultra-sonication for 30 min). The excess aqueous solution was removed from the Psf surface using a rubber roller before the surface was exposed to a solution of TMC (0.15 wt%) in hexane for 1 min, causing the creation of the thin dense PA layer through interfacial polymerisation **(**Fig. 3 **)**. The prepared TFNC membranes were rinsed with hexane and cured in an oven at 80 °C for 5 min, then soaked in DI water and stored in the fridge. The final membranes are named pTA-f-GO-x, with x representing the GO concentration; low (19 ppm), medium (38 ppm) and high (76 ppm), that was dispersed in the MPD solution prior to the interfacial polymerisation process.

### Membrane Characterisation

The membranes were characterised using techniques previously described. An Electrokinetic Analyser (Anton Paar, Austria) was used to measure the zetapotential and contact angle analysis was performed using the captive bubble technique using a Data Physics instrument (GmbH, Filderstadt, Germany)^24^ Air droplet images were taken and the contact angle analysed using OCA software. Transmission electron microscopy (FEI Tecnai G2 Spirit TEM) was used to analyse the morphologies of the prepared membrane cross-sections of the pristine and pTA-f-GO embedded membranes. Samples were immersed in white resin for at least one day before inserting them into gelatine capsules and then dried overnight at 60 °C. A Leica Ultra Cut ultra-microtome was used to obtain cross-sections of different membranes that were deposited onto copper grids covered with carbon/formvar (polyvinyl formal)^43^. The topography of the membrane surfaces were measured by AFM in non-contact mode via NT-MDT NTEGRA SPM^44^. Finally, the surface chemistry of the membranes were analyzed using a Nicolet Nexus 8700 FTIR Spectrophotometer (Thermo Electron Corporation) equipped with a ‘Smart Orbit’ ATR accessory and a Kratos Axis-Ultra DLD X-ray photoelectron spectrometer (Kratos Analytical, Manchester, UK) having a mono-chromated Al-Ka X-ray source and the obtained spectra were analyzed using CasaXPS software (Neal Fairly, UK)^26, 45^.

### Desalination performance of the membranes

The desalination performance of the membranes in terms of water permeation, salt rejection and chlorine resistance were performed using a high pressure stirred cell unit (Sterlitech HP 4750; effective membrane area 25 cm^2^), including digital balance and PC to collect data. An NaCl solution (2000 ppm) was used as the feed solution and tested under a trans-membrane pressure of 250 psi in all experiments. The water flux was evaluated by equation (3)^46^.

3 $${\rm{F}}={\rm{V}}/({\rm{A}}\times {\rm{T}})$$

where F, V, T and A represent the water flux (l/m^2^ h), volume of permeate (l), time (h) and the membrane area (m^2^), respectively. A Hach HQ 40 conductivity meter was used to determine the salt ion concentrations of the feed and permeate. R (%) was quantified via equation (4) in which c_1_ and c_2_ represent the salt concentrations of the feed and permeate, respectively.

4 $${\rm{R}}=(1-{{\rm{c}}}_{2}/{{\rm{c}}}_{1})100 \% $$

After 30 min filtration, the results were automatically logged by the digital balance. The average values of water flux and salt rejection were calculated from three separate experiments. The chlorine resistance capacity of the prepared membranes was performed by exposing membranes to sodium hypochlorite (NaOCl) solution (3000 ppm) for 1 h, prior to repeating the NaCl filtration test^37^. All statistical analyses were accomplished with the Minitab software v.17. One-way analysis of variance (ANOVA) was performed for various comparisons. Furthermore, Fisher’s Least Significant Difference (LSD) analysis was employed to designate the statistical significance. Results were displayed as a mean of three replicates and p value of 0.05.

### Biocidal properties of membranes

The antibacterial efficiency of membranes including pristine and pTA-f-GO fabricated membranes was quantitatively assessed according to the AATCC antibacterial test technique 100–2004 that measures the survival of *Escherichia coli* (ATCC 25922)^47^. All samples were cut to size (2.5 × 2.5 cm) and placed in sterile 6 well micro plates before the addition of a bacterial suspension (350 µL; 2 × 10^8^ CFU/mL) and incubation at 37 °C for 0 and 24 h. To quantify the number of bacterial cells, selected membranes were soaked in saline solution (25 mL-0.85% (w/v)) after the incubation time, and vigorously shaken for 1 min. The overall bacterial cell count was quantified by sequential dilution steps and pour plate technique by Luria Broth (LB) agar medium plates (10 g peptone, 5 g yeast extract, 10 g NaCl and distilled water up to 1 l; pH 7) incubated at 37 °C for 24 h. The biocidal efficiency of all samples was calculated using equation (5).

5 $${\rm{R}} \% \,=\frac{{C}_{0}-{C}_{{\rm{24}}}}{{C}_{0}}\times {\rm{100}}$$

where R is the bacterial reduction, C_0_ and C_24_ are the bacterial count immediately after inoculation and after incubation for 24 h, respectively.

## Electronic supplementary material

Supplementary Information 
