# Supplementary material for: A Novel Fabrication Approach for Multifunctional Graphene-based Thin Film Nano-composite Membranes with Enhanced Desalination and Antibacterial Characteristics
Source: Sci Rep. 2017 Aug 8;7:7490. doi: 10.1038/s41598-017-07531-y (PMC5548761; doi:10.1038/s41598-017-07531-y)
Supplement: Supplementary file 1 — Supplementary Information [file 41598_2017_7531_MOESM1_ESM.doc]

**Supporting Information**

**A Novel Fabrication Approach for Multifunctional Graphene-based Thin Film Nano-composite Membranes with Enhanced Desalination and Antibacterial Characteristics**

Hanaa M. Hegab^1,2^, Ahmed ElMekawy^3, 4^, Thomas G. Barclay^5^, Andrew Michelmore^6^, Linda Zou^1,7^, Dusan Losic^4^, Christopher P. Saint^1^, Milena Ginic-Markovic^6,*^

^1^ Natural & Built Environments Research Centre, University of South Australia, Adelaide, SA 5095, Australia

^2^ Institute of Advanced Technology and New Materials, City of Scientific Research and Technological Applications, Borg Elarab, Alexandria, Egypt

^3^ Genetic Engineering and Biotechnology Research Institute, University of Sadat City (USC), Sadat City, Egypt

^4^ School of Chemical Engineering, The University of Adelaide, Adelaide, SA 5005, Australia

^5^ Centre for Pharmaceutical Innovation and Development, University of South Australia, Adelaide, SA

^6^ Future Industries Institute, University of South Australia, Adelaide, SA 5095, Australia

^7^ Department of Chemical and Environmental Engineering, Masdar Institute of Science and Technology, Abu Dhabi, United Arab Emirates

*Corresponding. [milena.ginic-markovic@unisa.edu.au](mailto:milena.ginic-markovic@unisa.edu.au)

| **Table S1.** Analysis of variance (ANOVA) on-chlorinated/chlorinated membranes in terms of water flux and salt rejection. | | | | | | | |
| --- | --- | --- | --- | --- | --- | --- | --- |
|  | **Response** | **Source** | **DF** | **Adj SS** | **Adj MS** | **F-Value** | **P-Value** |
| **Non-chlorinated** | Flux | Membrane Type | 3 | 256.72 | 85.572 | 16.82 | 0.001 |
|  |  | Error | 8 | 40.71 | 5.089 |  |  |
|  |  | Total | 11 | 297.43 |  |  |  |
|  | Rejection | Membrane Type | 3 | 215.241 | 71.7471 | 19954.56 | 0.000 |
|  |  | Error | 8 | 0.029 | 0.0036 |  |  |
|  |  | Total | 11 | 215.270 |  |  |  |
| **Chlorinated** | Flux | Membrane Type | 3 | 62.58 | 20.860 | 3.91 | 0.055 |
|  |  | Error | 8 | 42.71 | 5.338 |  |  |
|  |  | Total | 11 | 105.29 |  |  |  |
|  | Rejection | Membrane Type | 3 | 543.980 | 181.327 | 5439.80 | 0.000 |
|  |  | Error | 8 | 0.267 | 0.033 |  |  |
|  |  | Total | 11 | 544.247 |  |  |  |

| **Table S2.** Fisher pairwise comparisons of non-chlorinated/chlorinated membranes in terms of water flux and salt rejection (95% confidence). | | | | | | | |
| --- | --- | --- | --- | --- | --- | --- | --- |
|  | **Response** | **Membrane Type** | **Mean** | **Grouping*** | | | |
| **Non-chlorinated** | Flux | pTA-f-GOL | 30.56 | A |  |  |  |
|  |  | pTA-f-GOM | 27.52 | A |  |  |  |
|  |  | pTA-f-GOH | 26.46 | A |  |  |  |
|  |  | Pristine | 18.08 |  | B |  |  |
|  | Rejection | pTA-f-GOL | 94.8289 | A |  |  |  |
|  |  | pTA-f-GOM | 92.3920 |  | B |  |  |
|  |  | Pristine | 87.2209 |  |  | C |  |
|  |  | pTA-f-GOH | 84.0365 |  |  |  | D |
| **Chlorinated** | Flux | pTA-f-GOM | 34.50 | A |  |  |  |
|  |  | pTA-f-GOL | 32.90 | A |  |  |  |
|  |  | pTA-f-GOH | 31.200 | A | B |  |  |
|  |  | Pristine | 28.33 |  | B |  |  |
|  | Rejection | pTA-f-GOL | 94.4000 | A |  |  |  |
|  |  | pTA-f-GOM | 90.767 |  | B |  |  |
|  |  | pTA-f-GOH | 82.700 |  |  | C |  |
|  |  | Pristine | 77.2000 |  |  |  | D |
| *Means that do not share a letter are significantly different. | | | | | | | |

| **Table S3.** Fisher pairwise comparisons of contact angle measurements with 95% confidence. | | | | | | |
| --- | --- | --- | --- | --- | --- | --- |
| **Response** | **Membrane Type** | **Mean** | **Grouping*** | | | |
| Contact Angle | Pristine | 47.10 | A |  |  |  |
|  | pTA-f-GOH | 31.08 |  | B |  |  |
|  | pTA-f-GOM | 28.50 |  |  | C |  |
|  | pTA-f-GOL | 22.29 |  |  |  | D |
| *Means that do not share a letter are significantly different. | | | | | | |
